# Aging: progressive decline in fitness due to the rising deleteriome adjusted by genetic, environmental, and stochastic processes

**DOI:** 10.1111/acel.12480

**Published:** 2016-04-06

**Authors:** Vadim N. Gladyshev

**Affiliations:** ^1^ Division of GeneticsDepartment of MedicineBrigham and Women's Hospital and Harvard Medical SchoolBostonMA02115USA

**Keywords:** Aging, Lifespan, Deleteriome, Theories of aging, Evolution

## Abstract

Different theories posit that aging is caused by molecular damage, genetic programs, continued development, hyperfunction, antagonistic pleiotropy alleles, mutations, trade‐offs, incomplete repair, etc. Here, I discuss that these ideas can be conceptually unified as they capture particular facets of aging, while being incomplete. Their respective deleterious effects impact fitness at different levels of biological organization, adjusting progression through aging, rather than causing it. Living is associated with a myriad of deleterious processes, both random and deterministic, which are caused by imperfectness, exhibit cumulative properties, and represent the indirect effects of biological functions at all levels, from simple molecules to systems. From this, I derive the deleteriome, which encompasses cumulative deleterious age‐related changes and represents the biological age. The organismal deleteriome consists of the deleteriomes of cells, organs, and systems, which change along roughly synchronized trajectories and may be assessed through biomarkers of aging. Aging is then a progressive decline in fitness due to the increasing deleteriome, adjusted by genetic, environmental, and stochastic processes. This model allows integration of diverse aging concepts, provides insights into the nature of aging, and suggests how lifespan may be adjusted during evolution and in experimental models.

## What is aging?

Aging remains a grand mystery of biology (Kirkwood & Austad, 2000; Vijg & Campisi, 2008). Numerous concepts have been advanced to define it, offering both evolutionary and mechanistic underpinnings, but none seem to explain it fully. Even the term ‘aging’ is interpreted differently among researchers, and the fundamental nature and the cause(s) of aging remain a hotly debated issue. The research community is essentially split among what seems to be incompatible ideas, with many scientists simply ignoring this very important biological question. Several theories on the nature and control of aging have contributed most significantly to this debate.

The *programmed theory* (Longo *et al*., 2005), built on the original 19th‐century insights of August Weismann, considers aging as a genetic program that has evolved to specifically direct senescence and death, thereby benefiting future generations. The term phenoptosis, similar to the programmed cell death term of apoptosis, was coined to describe such a program (Skulachev, 1997). However, while the undisputed role of genes in regulating aging does imply genetic, and therefore, program‐like features, there is currently no evidence of any gene or process that evolved specifically to stimulate aging or eliminate older individuals, and no mutants in any organism have been found in which such genes/processes are disrupted aborting the aging program. If the programmed aging theory is correct, why is mortality increasing from the beginning of early adulthood rather than late in life? A longer living organism can leave more offspring making it difficult to maintain the aging program. The idea of programmed aging as a universal aging mechanism also disagrees with the logic of evolution (e.g., How could selection bring about phenoptosis and preserve it during evolution, if the strength of natural selection declines with age?). While the program‐like nature of the aging process is hard to deny, in most cases the apparent ‘program’ may be more of a side effect of the main genes’ functions (which were selected during evolution). Thus, while some elements of the programmed aging theory seem logical, even if such a hypothetical program emerges, it is unclear how it can be maintained during evolution or how it can be universal in the biology of aging.

The *evolutionary theory* of aging, with its key concepts of mutation accumulation (MA) (Medawar, 1952) and antagonistic pleiotropy (AP) (Williams, 1957), founded on the original insights of Haldane (Haldane, 1941), suggests that the forces of natural selection decline as a function of age. This concept was formalized (Hamilton, 1966; Charlesworth, 1994) and examined experimentally (Rose, 1991). The theory posits that certain alleles could be selected for and mutations could accumulate in the genomes over evolutionary timescales, if these alleles and mutations show beneficial or neutral effects on fitness in early life, but are detrimental in later life when selection is inefficient to remove them. The MA concept does not constrain the pleiotropic effects of mutations across ages, whereas the AP concept suggests that the late‐acting detrimental alleles persist because they confer benefit at early ages. In essence, AP proposes that certain genes can influence two traits, a beneficial and a detrimental. Such genes will be selected if the first trait increases fitness in early life, even if the second trait is deleterious in later life. Therefore, deleterious alleles that act in late life will necessarily accumulate during evolution, causing aging. These insights suggest a scenario for how aging could evolve and imply that aging does not act for the good of species, that is, that aging is not programmed. The theory, however, supports program‐like features with regard to the role of genetics; for example, aging may be adjusted by AP genes. These great insights notwithstanding, the evolutionary theory is agnostic on the molecular mechanisms involved (completely different mechanisms could be accommodated by it, e.g., involving or not damage accumulation), leaves unsolved the identity and functions of genes and mutations that cause aging, requires that the AP genes have multiple effects/functions (which should also exhibit age‐specific, as well as both beneficial and detrimental effects), and is limited to organisms with clear separation of the germ line and soma (Williams, 1957) (essentially being limited to a subset of metazoa).

Another aging concept, the *free radical theory of aging* (Harman, 1956), offered an attractive mechanistic cause of aging, wherein reactive oxygen species generated as a consequence of metabolism randomly damage cellular components, with this damage gradually accumulating resulting in senescence. However, it is unclear what is so special about free radicals that cells cannot deal with them (e.g., by minimizing their production, repairing oxidative damage, or evolving better enzymes that do not make free radicals). From the evolutionary perspective, it is also unclear why oxidative damage would be worse than any other damage form. The same challenges apply to numerous other damage‐centric theories that focus on particular damage types, such as somatic DNA mutations, mitochondrial dysfunction, protein damage or aggregation, telomere shortening, and error‐prone biosynthetic processes. To accommodate these challenges, the free radical theory was extended to include other forms of damage (Orgel, 1973). However, this concept still could not explain why cells could not remove or repair this damage, or decrease its generation by evolving more efficient proteins. Overall, the free radical and other damage‐based theories offered attractive mechanistic ideas, but these were neither complete nor sufficiently linked with evolutionary biology.

The *disposable soma theory* (Kirkwood, 1977) further advanced the damage‐based aging by proposing the idea that organisms have limited resources that must be distributed between maintenance (e.g., processes that remove damage) and reproduction. The inability to allocate all resources (energy, building blocks) to maintenance (because organisms must invest into reproduction or they become extinct) makes protection less than 100% efficient, leading to damage accumulation. An important insight of the disposable soma theory is the fundamental role of trade‐offs in the biology of aging (Lemaître *et al*., 2014). A strength of this theory is also that it was able, for the first time, to integrate evolutionary and mechanistic biology of aging. On the other hand, it is unclear why resources should be universally limited (for all conditions and for all aging organisms) and why organisms with plenty of resources often live shorter than those with limited resources (e.g., calorie restricted) (O'Brien *et al*., 2008). The disposable soma theory also places emphasis on maintenance, even though other processes appear to contribute to aging as well. Thus, this attractive model also seems incomplete in describing the aging process.

A recently developed concept, the *hyperfunction theory of aging* (Blagosklonny, 2008), has also been able to integrate evolutionary and mechanistic biology, but replaced the disposable soma's ideas of molecular damage and resource allocation with excessive gene functions. It proposes that continued development and overactivity of genes in the reproductive age cause hypertrophy resulting in aging. Molecular damage on the other hand, even if it accumulates, is considered a bystander that has no influence on the aging process, or perhaps it represents a secondary factor (i.e., hyperfunction and hypertrophy cause damage, not the other way around). This thought‐provoking concept posits that the pathologies that lead to senescence are due to gene overactivity rather than damage, breakdown, and/or failure, and therefore, aging is viewed as an increasing mass of pathologies with different causes. This concept can be illustrated by the consequences of the excessive activities of MTOR and INS/IGF1 signaling. Inhibition of these pathways is predicted by the theory to increase lifespan, which indeed has been shown experimentally. But hypertrophy, at least in some cases, could also be the secondary (i.e., following damage) manifestation of aging or may represent other conceptually related deleterious processes in reflecting lack of protection or regulation. In addition, hyperfunction focuses on excessive activities, but there should also be activities that become insufficient during aging (e.g., insufficient activation of genes that deal with damage or incomplete replenishment of resources used throughout life) (de Magalhaes, 2012). The hyperfunction theory also does not explain how molecular damage, which undeniably accumulates with age, could be contained by cells or considered irrelevant if excessive gene activities are key to aging. For example, if hyperfunction causes aging, selection should be relaxed on genes and processes that produce damage (relative to those involved in hyperfunction), so they will produce more damage. Eventually, the impact of damage will increase, roughly synchronizing with that of continued development, so both hyperfunction and molecular damage should then causally contribute to aging.

It is clear that while the aging theories are very different, each of them touches a particular aspect of the aging process and, within that context, has merit. However, because these concepts operate at different levels of biological organization or limit themselves to particular biological processes or molecular components, they point to different manifestations of aging. It is undeniable that program‐like features, increased molecular damage, excessive biological functions, deleterious effects of AP alleles and mutations, trade‐offs, etc. occur during aging, but which one is primary? I argue below that the existing theories of aging can be integrated into a concept, which utilizes particular aspects of each theory, brings new ideas, and completes the gaps.

## Imperfectness as a basis of aging

Physicochemical principles dictate that all biological molecules and all biological processes are imperfect (Gladyshev, 2013). For example, proteins, in addition to their direct functions (i.e., functions they evolved for), engage in unwanted reactions and interactions. Enzymes are characterized by the nonzero likelihood to react with other substrates, generating minor reaction products (Golubev, 1996; Gladyshev, 2012). Concentrations of components of various protein complexes are not perfectly adjusted, resulting in deleterious consequences. Replication, transcription, and translation are well known to be error‐prone processes. Large genomes are full of mildly deleterious variants and other signs of inefficient selection, such as repeats and mobile elements, but even very small genomes contain elements of increased genomic entropy. Genetic drift imposes an additional constraint on the level of genetic perfection achievable by biological systems in finite populations. Fluctuations and drifts in gene expression and metabolite levels are pervasive and, together with other processes, contribute to inherent noise, infidelity, and heterogeneity associated with cellular life. Regulation is imperfect too, and all these are exacerbated during the aging process. In fact, imperfections penetrate the entire biology and chemistry of organisms, leading to unwanted, deleterious, disordering, damaging consequences at all levels, from simple molecules to organs.

This damage has both stochastic (based on physicochemical principles) and deterministic (resulting from specific genes and genetic programs of a particular cell/organism and defined environmental conditions) components. At greater evolutionary distances, the effect of genetics on aging is strong. For example, a human, a dog, and a mouse exposed to the same environment (e.g., living in the same house) will have widely different mean lifespans, so this information must be encoded in their genomes. However, differences within homogenous populations are best explained by stochastic and environmental components of damage. For example, genetically identical animals, when exposed to the same environment, exhibit significant variation in lifespan; this variation is largely due to stochastic processes. What is the basis for the nonrandom (deterministic) damage due to genetic factors? Genome‐encoded enzymes will make specific forms of damage rather than any damage (because they are built to deal with particular substrates but are imperfect), and proteins and RNAs will show preference for unwanted interactions with particular cellular components rather than any components. Therefore, cumulative damage will be roughly the same for individual organisms within species, defining maximal lifespan of this species. This notion also implies that much of the damage is indirectly encoded in the genomes through the genes that make this damage. This damage will change during evolution (because the genomes that encode the molecules that make this damage change), as well as during the organism's life (because the damage will accumulate as a function of age).

Because the genome, its every gene and all processes utilized by an organism are imperfect, all producing damage at one level or another, the resulting diversity of damage forms will be too numerous to be dealt with by nondividing cells, regardless of how evolution shaped the organism. Moreover, protective functions against this damage will themselves be imperfect and produce other forms of damage. What postmitotic cells/organisms can do then is to deal with the most deleterious forms of damage that otherwise severely affect fitness, leaving milder damage forms aside. The latter will therefore accumulate over time. Most of these damage forms will simply be invisible to evolutionary processes, as selection can never be strong enough to deal with all damage. For example, if five molecules of a particular nontoxic by‐product are produced during the lifespan of a cell, no enzyme will evolve to protect against this damage. A billion types of such five‐molecule damage forms will likewise be invisible to selection, but together they will contribute to aging. In other words, mild damage is the damage that can be tolerated by organisms until late life, when reproduction has been accomplished. Because it is cumulatively deleterious and accumulates gradually, its manifestations also emerge gradually, for example, they may be reflected in the gradual increase in mortality starting from early adulthood. These considerations imply mutually reciprocal, cumulative causation of aging, with contributions from all processes, even though these contributions are widely different, both in form and impact.

Mild damage represents the well‐known age‐related manifestations of the aging process, but is not limited to damage in the classical sense (e.g., damage to proteins, lipids, nucleic acids, metabolites) and encompasses any age‐related deleterious change. For example, if a cofactor accumulates in a short‐lived organism during embryogenesis and is slowly used during adulthood, the organism may lose the corresponding biosynthetic enzymes during evolution; the gradually decreased levels of this cofactor during the life of this organism would represent mild damage, which can be tolerated until after the organism reproduces. Loss of regulation is another example. If the entire amount of a metabolite generated by a gene product is used throughout a reproductive lifespan, a regulatory system to switch this gene off may not be maintained. Therefore, in the postreproductive period, the gene will continue to produce this product, which will be an unwanted deleterious age‐related change, akin to those described by the hyperfunction theory. Similar analogies apply to increasing or decreasing levels of metabolites, changes in gene expression, protein synthesis, and cell growth among others. Such age‐related changes may be expected to show unidirectional or bell‐shaped changes starting from the beginning of development to late life, which indeed has been demonstrated for gene expression in primates (Somel *et al*., 2010). The processes that are mildly deleterious may nevertheless be represented by obvious phenotypic changes. For example, mammals may accumulate fat, become bald, or feature other well‐known aging phenotypes, all of which are also the manifestations of mild damage—the tolerated deleterious processes. Altogether, imperfections of biomolecules and bioprocesses will inevitably lead to deleterious age‐related changes, encompassing molecular damage and other processes, which cumulatively manifest as aging.

What is discussed above in this section applies to postmitotic cells and organisms. However, the myriad of mild damage forms generated in any cell or organism does not stop germ line maintenance, because damage of the germ line is always diluted by cell division. To some degree, cell division allows damage dilution in somatic cells as well, which is further extended by the use of stem cells (to replace damaged and senescent cells), synthesis of daughter cells from within mother cells, exocytosis, and by other mechanisms. Damage dilution by cell division is sufficient to handle all damage forms except mutations and, to some degree, epimutations, whereas all other mechanisms could only deal with certain damage forms. Conceptually, damage dilution in somatic cells (especially postmitotic cells) is also similar to increased redundancy of the system (e.g., many cells of the same type in an organ), as in the reliability model of aging (Gavrilov & Gavrilova, 2001). Damage dilution is common in unicellular organisms that divide symmetrically. This is a key strategy that allows cells to deal only with some damage types, whereas the unabundant mild damage is simply diluted when cells divide (Ackermann *et al*., 2007; Evans & Steinsaltz, 2007; Gladyshev, 2012). Damage dilution is also possible in organisms, in which adult stem cells can generate all cells in the body, for example, in hydra and planarians. However, if an organism has postmitotic nonrenewable cells (e.g., neurons), aging is theoretically unavoidable, even though lifespan could be very different depending on organism.

Therefore, the fundamental nature of aging emerges even before evolutionary processes can come to play. The true root cause of aging is imperfectness, which is something that is not selected during evolution. It is a property of matter and, therefore, of all biomolecules that make up a living organism. Imperfectness is also at the heart of life, because it produces variation from which more fit organisms can be selected. It may be said that imperfectness defines both the origin of life and the origin of aging, whereas adjustments to the degree of imperfectness define adjustments in the aging process, such as control of lifespan. Any factor that adjusts the aging process (e.g., environment, genes, mutations, AP alleles, stochastic processes) often has nothing to do with the cause of aging. Consider a metaphor of a river, where lifespan is the time needed for the water to flow from the mountain to the ocean (Gladyshev, 2012). Freezing the river into a glacier, routing it to a more steep or flat terrain, or building a dam can increase this time, which is analogous to altering lifespan. However, these manipulations do not tell us about why the water flows along the river. It flows because of gravity. In aging, gravity is the equivalent of imperfectness, whereas adjustments that affect the flow of water represent the various factors that affect longevity. The cause of aging is fundamentally different from the determinants of longevity.

## The deleteriome

It should be apparent from the text above that damage, a term often used in aging research, is somewhat limiting, because the proposed model attempts to encompass all age‐related deleterious processes—not only damage in its classical meaning (i.e., molecular damage to cellular components), but also lack of (or excess of) the proper control of biological processes, such as deleterious changes resulting from variance in gene expression and metabolic remodeling. Another aspect of deleterious processes is represented by hyperfunction. Consider yolk steatosis in *C. elegans* (Ackerman & Gems, 2012): Continued yolk production after completion of reproduction is not molecular damage in the classical meaning of this term. At a further level of complexity, changes during the aging process are represented by an increased disorder of the system.

It is then useful to define the deleterious effects of all these processes as the deleteriome. This term encompasses molecular damage, consequences of additional deleterious processes, as well as increased disorder at all levels, from simple molecules to cells and organs. It is cumulative, gradually increases as a function of postreproductive age, and is deleterious to organisms. The deleteriome increases with the postdevelopmental age of organisms in a quasi‐programmed way, and is ultimately defined by genetic, environmental, and stochastic processes (Box 1). Its individual components influence each other in a mutual, reciprocal manner, together increasing disorder of the system, and manifesting as aging. The deleteriome reflects the total work done by the system (the sum of all activities), but not the total energy expenditure or the total resources available. The rising deleteriome is the true meaning of the aging process caused by imperfectness and adjusted by numerous factors acting in concert.

Box 1 **Genetic, environmental, and stochastic factors as main drivers of lifespan variation.**

Contributions of various factors to biological aging can be illustrated by the metaphor of an aging car. Here, the length of an organismal lifespan is analogous to the mileage driven over the car's lifespan. It is influenced by the make/model of the car (analogous to the effects of genetics) and road conditions, weather, and fuel quality (representing the effects of environment). Better built cars, like better road conditions, milder weather, and better fuel, will be associated with longevity. In addition, random processes influence lifespan. These stochastic events include internal processes of the car leading to damage accumulation, gradually increasing the chance the car breaks, as well as random events associated with driving (stopping, accelerating, turning, accidents, etc.). For example, a car driven on highways is expected to accrue more miles than when it is driven in city. Likewise, biological aging is influenced by genetics, which is a major contributor when aging is considered across species and genetically heterogeneous populations, environment, and stochastic processes.

As the deleteriome consists of diverse forms of damage and other deleterious processes, it is currently not accessible in its entirety. Difficulty in measurement notwithstanding, the deleteriome may be viewed as a measure of biological age of the cell, organ, or system. This implies that the best markers of aging would be the measures of the deleteriome. Such markers have not been well defined, as the focus of previous research has been on particular age‐related changes, such as telomere length, oxidative damage, and expression of a limited number of genes. But such limited assays would be misleading in representing organismal aging and comparison across organisms and cell types. However, recent research shows that the candidate markers that best represent the deleteriome, because they include measurements of many diverse age‐related parameters simultaneously, for example, genomewide epigenetic changes, mutations, nontargeted metabolite profiling and gene expression (Hannum *et al*., 2013; Horvath, 2013; Avanesov *et al*., 2014; Hoffman *et al*., 2014), offer the best predictive models of the progression through aging. For example, the DNA methylation clock based on multiple CpG sites was found to accurately predict human age (Hannum *et al*., 2013; Horvath, 2013). The changing DNA methylome defined by the clock may be viewed as a subset of the deleteriome. There is every expectation that the already impressive accuracy of estimation of the biological age (but not chronological age, as organisms age differently due to genetic, stochastic, and environmental factors) may be further improved by better representing the deleteriome through age‐related deleterious changes and building more sophisticated computational models.

Further complexity arises from the fact that different cells within organs, and organs themselves, age with different rates, and these rates are unequally affected by the environment. Therefore, the biological age of an organism is difficult to determine by analyzing biomarkers of individual organs. As the organismal deleteriome is defined by the deleteriomes of cells and organs, each changing according to their own roughly synchronized trajectories, we face similar challenges of quantifying it. Ideally, all organs and systems would need to be monitored for their deleteriomes, and this should be done as a function of the genotype and environment. Despite these challenges, the direct link between the deleteriome, biomarkers of aging, and the biological age at every level within the organism, and at the whole organism level, allows defining the aging process at the molecular level and offers an opportunity to quantify it.

## How does aging begin?

There are organisms that age, and there are those that do not, but distinction between the two is not always obvious. For example, most animals and plants, the budding yeast, and some asymmetrically dividing microorganisms are known to age, whereas symmetrically dividing microorganisms (generate identical cells) and some animals lacking nonrenewable cells (certain planaria, hydra, and other organisms whose adult stem cells can generate any cell type) are in the nonaging category. By simply considering the phylogeny of organisms that age and those that do not, it is clear that aging evolved at least several times independently. Starting from protocells, the earliest life forms, symmetrically dividing nonaging cells occasionally transitioned to asymmetric division, initiating aging in this species. It also appears that some aging organisms gave rise to nonaging ones. To understand the origin of aging, it is important to consider how these transitions occur. In this regard, an interesting observation was made that some extant organisms exist in both aging and nonaging modes. For example, *S. pombe* normally divides symmetrically and does not age, but under stress one of the daughter cells selectively inherits more damage, grows in size, and eventually dies, whereas the other cell is cleared of this damage, effectively being rejuvenated, and may proceed with symmetrical division (Coelho *et al*., 2013). Conditional aging was also described for *E. coli* and other organisms (Watve *et al*., 2006). Thus, stress, or more broadly changing environment, is an important factor in the origin of aging.

Examples of organisms that age, and those that do not, illustrate the fact that the classical evolutionary theory of aging does not apply to all aging organisms, consistent with the original insight (Williams, 1957). Having the same genes and mutations, *S. pombe* can be classified as both aging and nonaging organisms. Moreover, any symmetrically dividing nonaging microorganism will age if its cell division is blocked, because damage will accumulate in it regardless of whether the aging‐causing alleles have time to accumulate. Senescence of immortal cell lines in mammals is another example of such nonaging to aging transition, whereas immortalization of somatic cells is an example of the transition from aging to nonaging states.

Generalizing these observations, it seems conversion from nonaging to aging states can be sudden. It may be sufficient for an organism to shift to asymmetric division, or start using nonrenewable cells, or employ some other innovations, to transition from nonaging to aging states. Everything that is needed for aging to occur is already present in a symmetrically dividing nonaging organism.

As discussed above, since all purposely used biomolecules, including genes, are imperfect, they contribute to the rising deleteriome. These properties are built in and represent a fundamental nature of these molecules. Additional mutations may evolve over time, and in some cases, this may lead to the conversion from nonaging to aging organisms, but this does not apply universally and therefore does not support inevitability of aging. Once a species transitions to an aging state, additional alleles can play a role in altering the trajectories along which the deleteriome accumulates, in turn leading to different lifespans and associated life‐history traits across species.

It has been unclear how the evolutionary theory of aging deals with the fact that some organisms do not show increased mortality and decreased reproduction with age and that in some cases (tortoises, fishes), they show decreased mortality and increased reproduction (Jones *et al*., 2014). This seems to disagree with the prediction that, because the strength of natural selection declines with age, mortality should increase and reproduction decrease.

Dobzhansky once famously stated that ‘nothing in biology makes sense except in the light of evolution’. Although debatable, the nature of aging, its root cause built on imperfectness, is the one thing that may happen outside of the realm of evolutionary biology. From the perspective of the deleteriome model, imperfectness, ecology, and constraints associated with the biology and organization of the organism define whether this organism ages or not, genomes largely define species lifespan, and genetic variation, environment, and stochastics define difference in lifespan at the population level.

## How is lifespan adjusted?

Generation of age‐related deleterious changes is influenced by genetic, environmental, and stochastic processes, which vary in their contributions to the deleteriome depending on the genetic program of an organism and the conditions in which it occurs (Box 1). Various deleterious forms generated as a result of these processes should unequally contribute to the aging process, but none would be expected to be the major damage form that limits lifespan. If an organism suffers from a major aging‐contributing damage, selection may be relaxed on other deleterious forms (although differently depending on whether the damage forms contribute to the deleteriome and its components additively, epistatically, or independently), and their damaging effects will then increase until they approach the deleteriousness of the original major damage. In the end, many damage forms will contribute to the aging process and do so in a cumulative manner.

Although many damage types have been implicated in the aging process, none has been demonstrated to be necessary for aging to occur. This idea can be illustrated by the role of DNA damage in aging. Although studies have shown that mutations increase with age in every organism tested and that mutations in many DNA repair genes reduce lifespan, it was recently demonstrated that the impact of mutations alone is too small to cause aging in budding yeast (Kaya *et al*., 2015). Although most yeast cells did not have any mutations toward the end of their life, they aged and died. Therefore, while DNA damage contributes to cumulative damage, it cannot be a single, or even a major, contributor. Thus far, it has not been possible to measure the impact of other individual damage types on aging, but the example of the well‐studied oxidative damage (Gladyshev, 2014) suggests that what was found for mutations should also apply to other forms of damage.

The notion of the lack of primary damage forms (or primary deleterious age‐related changes) in organisms and rough synchronization of deleterious forms in their impact on aging implies that many processes that contribute to the deleteriome need to be coordinately adjusted in order to adjust species maximal lifespan, a point also argued by evolutionary biologists (Rose, 1991). This creates a conundrum, as lifespan can be changed dramatically during evolution; within species, it can even be changed by single‐gene manipulations. This can be explained, however, by the fact that the major regulators of longevity are the genes that affect many other genes and processes, that is, those that globally affect cellular metabolism, and therefore the deleteriome. For example, altered activities of GHR, IGF1, and MTOR may change fluxes through major energy‐generating and energy‐utilizing pathways. These are some of the currently best‐known genes whose deficiency increases lifespan. Nature may also utilize these lifespan ‘regulating’ strategies, for example, by altering thermogenesis in naked mole rats (Fang *et al*., 2014) and the GH/IGF1 axis in microbats (Seim *et al*., 2013), both of which are exceptionally long‐lived mammals. The increased lifespan can also be achieved by slowing down metabolism, for example, by decreasing environmental temperature (for exothermic organisms), a well‐known lifespan extending procedure.

Evolutionary experiments in model organisms made clear that laboratory selection could rapidly change lifespan. For example, classical experiments in fruit flies showed that selection for early and late reproduction could decrease and increase lifespan, respectively (Rose, 1991). In addition, screens in model organisms revealed that inactivation of many single genes may increase lifespan, although such changes in longevity may come at the expense of fitness. Thus, evolution appears to adjust multiple genes and in various combinations, which cumulatively impact species longevity. In other words, there are many roads to longevity. Adjustments in lifespan, like adjustments in other life histories, are driven mainly by evolutionary processes, for example, in response to changes in environment (Stearns, 1992). This is an active area of research, as theoretical considerations may be examined against experiments. An attractive possibility is also that the evolution of longevity is related to the evolution of complexity, which proceeds through mostly neutral or slightly deleterious mutations as a result of the small effective population size (Koonin, 2011). Longevity does not equate evolutionary success (Box 2), but understanding the genetic strategies that lead to changes in species lifespan (Fushan *et al*., 2015; Ma *et al*., 2015) may help apply them to human aging.

Box 2 **Is longevity an evolutionary success?**

It is often discussed that long‐lived organisms, on the grand scale of things, are more successful. Indeed, they are typically larger, more complex, and more intelligent than the short‐lived organisms. But from an evolutionary perspective, the success is defined differently and may often look like the opposite of being big and smart. First, lifespan is unlikely to be a major trait selected during evolution, as evolution works by maximizing fitness. Second, shorter‐lived species may be viewed as more fit if they can leave more progeny, develop faster, and sustain population growth. This is also reflected in their genomes, which are simplified and often have fewer introns (in both number and length), repeats, mobile elements, and other ‘junk’ DNA forms. Longer‐lived organisms typically have smaller effective population sizes, implying that they evolve primarily through nearly neutral mutations, that selection is less efficient in these organisms, and that they cannot effectively eliminate deleterious alleles.

Although the rising deleteriome is the basis for aging, organisms most often die from disease rather than aging. In essence, aging may be viewed as a combination of age‐related diseases. Deleterious consequences of cellular functions are synchronized only roughly, because genetic, environmental, and stochastic processes adjust aging trajectories within a population (Box 1). Age‐related diseases may be particularly pronounced when they result from deviations from perfect synchronization of deleterious processes. Perfect synchronization would have led to a situation akin to the Oliver Wendell Holmes’ ‘one‐hoss shay’, which was built to last exactly 100 years and then went to ‘pieces all at once, and nothing first’. But this is not observed in biology. Most people die from particular age‐related diseases, such as cancer and heart disease, exposing deviations from synchronized aging due to genetic predisposition, environmental factors, and stochasticity. This underscores the intimate relationship between aging and age‐related diseases.

## Intersections of the deleteriome model with other theories of aging

I will now discuss how the model described above can link the previously proposed aging theories and offer a more integrative view on aging. First, the fact that much of the deleteriome is nonrandom and indirectly encoded in the genome (through the genes that contribute to these changes) explains the program‐like features of aging. However, it is not truly programmed, that is, there are no genes that evolved specifically for the purpose of aging (there is no such thing as the survival of the unfittest). Instead, all genes indirectly contribute to the deleteriome, and what appears as the aging program is in fact the program of life. This program both stochastically and deterministically elevates the deleteriome through indirect functions of its components (i.e., deleterious functions for which they have not evolved). It is also consistent with gradual changes during the aging process and mortality increases starting from an early reproductive age. So, aging emerges as a quasi‐program, a feature that links the proposed model and the *programmed theory of aging*.

The proposed model also encompasses the *hyperfunction concept*. Here, continued development and gene overactivity, like mild damage and other slightly deleterious processes, represent deleterious age‐related changes, from which there is no protection, reflecting a lack of selective advantage that such a mechanism would confer. Several characteristic examples of hyperfunction have been described. For example, nematodes continuously generate yolk, even after they stop reproducing, leading to yolk accumulation in old animals—the yolk steatosis (Ackerman & Gems, 2012). It was proposed that this represents organ dysfunction due to hyperfunction. Indeed, the origin of the yolk steatosis pathology is the normal yolk synthesis rather than damage accumulation. Nematodes are unable to halt yolk production because its deleterious effect becomes evident only in old animals, after reproduction is accomplished. Conceptually, yolk accumulation does not differ from the accumulation of molecular damage, for example, carbonylated proteins or lipofuscin, which are well‐known examples of the damage implicated in the aging process in mammals (Yin, 2016). Thus, both yolk steatosis and molecular damage represent mild deleterious age‐related changes and contribute to the deleteriome. They are not mutually exclusive, can be adjusted by the same evolutionary processes, and ultimately have the same basis, imperfectness.

An additional example is yeast cells accumulating extrachromosomal ribosomal DNA circles, which was suggested to be a cause of aging in this organism (Sinclair & Guarente, 1997). The proposed model would consider the DNA circles as a form of mild damage and a component of the deleteriome. Another example is a steady loss of certain lipids in fruit flies. Flies appear to be born with the lipid reserves that are incompletely replenished during their adult life (Avanesov *et al*., 2014). Here, the deleteriome is represented by the lack of certain lipids in later life. Both nematodes and fruit flies feature gradual global changes in gene expression and metabolite levels (Budovskaya *et al*., 2008; Avanesov *et al*., 2014; Hoffman *et al*., 2014), which may also be viewed as mild deleterious changes, akin to molecular damage. At the gene level, excessive activity of MTOR was proposed to be responsible for the aging process (Blagosklonny, 2008), which is another example of the deleteriome. Like too high or too low activities of other genes, MTOR's excessive activity represents age‐related mild deleterious changes.

The idea of hyperfunction was illustrated by the metaphor of workers, who are given instructions to build a house, but no instructions to stop construction when the house is built (de Magalhaes, 2012). By analogy to continued development during adulthood, the workers continue to add new layers of carpet in the completed house, paint walls over and over, and keep on roofing until the house becomes nonfunctional and collapses. From the deleteriome perspective, the continued construction‐inflicted dysfunction of the house would be roughly synchronized with the damage from other processes (environmental, wear and tear from extensive use, infrastructural, plumbing and electrical damage, errors made by workers during continuous construction, etc.). Overall, the hyperfunction concept can be well integrated with the deleteriome model proposed, as well as with the evolutionary theory, wherein biological imperfectness is the cause of aging, and gene overactivity, like molecular damage, is one of its manifestations, representing cumulative deleterious age‐related changes adjusted and synchronized by evolutionary processes.

An additional manifestation of imperfectness is hypofunction (it is conceptually analogous, but opposite, to hyperfunction, although this term has not been previously discussed in the literature), representing insufficient activity during aging (de Magalhaes, 2012). Hypofunction may arise when certain functions cannot be sufficiently activated or switched on. An opposite to the example of yolk steatosis (where there is no selective advantage to switch off yolk synthesis during aging), there is no selective advantage to switch on genes acting on mild damage, such as repair and detoxification proteins. In a more extreme case, such genes (those that act in late life) may simply be absent. Overall, both hyperfunction and hypofunction concepts, representing subsets of deleterious age‐related changes, integrate well with the proposed model.

Genes generally benefit organisms and are selected for their functions. However, the same genes also contribute to cumulative deleterious changes, which increase throughout an organism's life, so the beneficial direct functions of genes will be overcome by their adverse indirect effects in later life. The *evolutionary theory of aging*, with its concepts of AP and MA (Medawar, 1952, Williams, 1957), proposed that certain alleles may exert both beneficial effects (or neutral in the case of MA) on some traits in early life and detrimental effects on other traits in late life, from which scientists derived the existence of aging and explained how it evolves. These concepts have been of fundamental importance for understanding the aging process. However, they have not led to insights into the molecular mechanisms and have not resulted in the characterization of specific alleles and mutations that cause aging (although some genes such as IGF1 and MTOR may qualify as AP genes). The tests for early and late reproduction as well as tests of mortality patterns claimed to support the evolutionary theory (Rose, 1991), but these tests are also compatible with other concepts of aging. Deficiencies of the classical models or their mathematical treatment have also been recognized by other scientists (Moorad & Promislow, 2008; Jones *et al*., 2014; Wensink *et al*., 2014).

The notion that a pleiotropic allele influences two antagonistic traits ‘equates’ these traits. I suggest that there is a key difference between the beneficial trait, which is the trait selected, and the detrimental trait, which is nothing else but the unavoidable deleterious consequence of the beneficial trait. Selection can adjust such deleterious effects, but typically does not originate them. Their origin is imperfectness. Recall the river metaphor discussed above (Gladyshev, 2012): Imperfectness is the equivalent of gravity, which is the reason the river flows, whereas the evolution of lifespan relates to the factors that affect the flow of water. In addition, while AP offers insights into how genes can be selected to influence traits in contrasting ways, the molecular basis of such processes and how they integrate with other cellular processes, which are also selected, remained obscure. What are the AP alleles/genes? What are their functions? How many of them are present in a genome? If aging is directed by certain pleiotropic genes, do other, nonpleiotropic genes influence the aging process? If so, how? The evolutionary theory of aging has not given satisfactory answers to these questions or is simply agnostic on these issues. However, the notion that AP is not between the two antagonistic traits, but between direct and indirect functions of genes immediately addresses these questions. The beneficial function is selected during evolution, while the deleterious one (i.e., errors of all sorts, nonspecific interactions and other consequences of imperfectness that comprise the deleteriome) is not. Because all genes are imperfect, all genes are AP‐like genes; therefore, the search for AP vs. non‐AP genes becomes meaningless.

The evolutionary theory of aging also proposes that there are limits to perfection because of the existence of AP alleles and mutations. But imperfect genes are just a subset of imperfect biology, as perfection is impossible to accomplish at any level of biological organization, not only at the level of alleles/genes/mutations. All purposefully used molecules (e.g., metabolites, trace elements, cofactors) have AP‐like properties. Organisms use these molecules because they offer benefits, but imperfectness leads to an accumulation of deleterious changes due to the use of these molecules. It is often discussed that deleterious effects are late‐acting; however, in the context of the deleteriome, it would be more accurate to state that the deleterious effects are cumulative rather than late‐acting.

As discussed above, it is also unclear how the evolutionary biology of aging explains the existence of apparently nonaging organisms (Finch, 1990), such as hydra and planarians, as well as organisms in which mortality decreases and fecundity increases with age, whereas this is easily explained by my proposed model. Hypothetically speaking, one could try to genetically engineer an organism devoid of all AP alleles and Medawar's mutations, thereby making this organism nonaging. From the deleteriome standpoint, this attempt is futile, not only because perfect genes cannot be made, but because other biomolecules also produce cumulative deleterious changes. Again, imperfect genes are just one of the many manifestations of imperfect biology. In the end, it seems that AP is not necessary and it may not be sufficient to cause aging, because the AP‐less organisms would still age, and because certain organisms with AP alleles and mutations may not age. It appears that the insights into aging from an analysis of evolutionary forces can inform us on how organisms age, but not necessarily why they age. Aging seems inevitable even before specific evolutionary considerations come into play. It is important to stress that the indirect effects of biological functions represent age‐related deleterious changes and embody the deleterious effects of AP alleles and mutations, whereas the direct, evolved functions embody the beneficial effects. This simple idea resolves the nature of the pleiotropic effects that are at the core of the evolutionary theory of aging, offers a molecular insight, and extends the evolutionary concepts to all genes and all purposely used biomolecules.

The proposed model also intersects with the *disposable soma theory*. For example, the trade‐off between reproduction and maintenance, a central insight of the disposable soma theory, can be explained by the fact that reproduction is metabolically demanding, so it will increase cumulative damage, thereby shortening lifespan. Therefore, the key here is not allocation of limited resources *per se*, as proposed by the disposable soma theory, but the limited capacity for total cellular activity, because activity leads to elevation of the deleteriome. It is also not fully clear what is meant by the limited resources in the context of this theory. If this includes everything needed for organisms to thrive (nutrients, energy), then one can imagine a situation when such resources are available to species in excess for many generations. Thus, in the context of aging, the molecular basis of trade‐offs is not the limited resources, but the deleteriome the use of these resources increases.

The proposed model also explains why maintenance cannot be 100% efficient. It is not only because of trade‐offs, but because all processes, including maintenance, are imperfect and because there are more damage forms than protective systems. In other words, efficiency of maintenance cannot reach 100% even if all resources are used for it. Imperfect repair is an example of a process that leads to tolerable, mild damage. Repair brings severe damage to the level of mild damage, which cumulatively contributes to aging together with other mild damage forms, from which there is no protection. Damage dilution by cell division and cell renewal are the strategies that allow reaching a balance between damage generation and removal (but this is not applicable to postmitotic nonrenewable cells, so organisms with such cells will necessarily age). Thus, the proposed model redefines the disposable soma theory and links it naturally with both classical evolutionary concepts and quasi‐programmed ideas.

*Oxidative damage* and other damage forms that are increased during an organism's life also represent mild damage, which can be tolerated until the postreproductive period, and the intersection with the theories that focus on other individual damage forms (i.e., telomere shortening, errors in protein synthesis) is similarly obvious. None of these damage forms can be viewed as a major damage form, but they all contribute to cumulative deleterious age‐related changes, that is, to the deleteriome.

The proposed nine *hallmarks of aging* (López‐Otín *et al*., 2013) describe some of the phenotypes associated with the patterns of the deleteriome change with age. These hallmarks, while useful to generalize features of aging, do not represent the causes of aging. Additional hallmarks can also be defined, such as numerous examples of deregulated processes, dysfunctional cellular compartments, and changes in gene expression. All these features should be viewed as a whole, as their relative importance may be changing depending on organism, cell type, conditions, and diet (e.g., telomere attrition only applies to dividing cells and epigenome changes and cell communication only apply to organisms with separated soma and germ line).

Overall, with regard to the contribution of various mechanisms to the aging process, there cannot be a major universal contributor, and the previous theories do not represent the ultimate cause of aging. However, all the mechanisms invoked by these theories of aging do contribute to the deleteriome. These contributions include, but are not limited to, mutations with delayed deleterious effects (mutation accumulation concept), alleles which increase fitness in young organisms but have deleterious effects in late life (antagonistic pleiotropy concept), occurrence of age‐related mutations (the concept of mutations as the cause of aging), oxidative damage (free radical theory of aging), trade‐offs and incomplete maintenance (disposable soma theory), and continued developmental processes, including overactivity (hyperfunction concept) and underactivity (hypofunction concept).

## Concluding comments

Many have little appreciation for defining the cause(s) of aging and distinguishing them from the control of lifespan (Box 3). My hope is that, after reading this piece, some scientists may take another look at this issue. If a researcher tries to understand aging by studying a particular favorite gene or process (sometimes chosen as much for personal or political reason as for scientific), rethinking of causal effects in aging may change the way this research is done or interpreted. Not only is the focus on single genes and processes often misleading or even outright incorrect, it profoundly limits understanding of aging as a whole. As discussed above, if there is a single process that limits lifespan, selection may be relaxed on other genes and processes, so their impact on aging should increase until it partially synchronizes with that of the original limiting process. In the end, the impact will necessarily be cumulative and roughly synchronized, with contribution from all genes and processes involved instead of being single gene‐ or process‐driven.

Box 3 **Do we need a general concept of aging?**

Many researchers think that a general concept of aging is not needed or are simply agnostic on this issue. In part, this is because there are already so many ideas in the field, which are difficult to test or even relate to each other. This author disagrees with this logic. Nothing can be more important than the universal concept of aging to guide future research. Groundbreaking ideas, such as the role of proton gradient in ATP synthesis, the periodic table of chemical elements, and the evolution by natural selection, which were the concepts not proven at the time of their proposal, are good examples of how broadly reaching ideas on important biological questions can influence future research.

A similar issue is with the models that assign causal roles to a particular, even if broadly important process. Why do we adapt such a narrow focused view on aging? Each aging theory seems to describe a particular aspect of aging, but they are also incomplete in other ways. Is there a program of aging? Yes or no. The program of aging (or rather a quasi‐program) does exist, but it includes all genes, and its purpose is life, not death. This is a program of living, whose by‐product is aging. Do certain late‐acting mutations and AP alleles exist that contribute to aging? Yes, but these mutations and alleles are typically a matter of lifespan adjustment. All genes have these properties (including those in nonaging organisms), and not only genes, but also all purposely used biomolecules and biological processes. Is the force of natural selection lower in late life in age‐structured populations? Yes, but this does not necessarily lead to typical aging patterns. Do ROS, DNA damage, various error‐prone processes, etc. contribute to aging? Yes, but none serve as the major contributor, because they act cumulatively. Are repair processes less than 100% efficient, consistent with the disposable soma ideas? Yes, but not only repair, all processes are imperfect, and the focus on repair, like the focus on individual damage forms, is limiting. Do trade‐offs exist? Yes, but in the context of aging, they are defined by the deleteriome and not by the limited resources. Do continued development and hyperfunction play a role in aging? Yes, but the inability to stop or regulate processes in late life is fundamentally similar to the lack of protection against other deleterious processes, such as molecular damage. Like common damage forms that accumulate during aging, hyperfunction (and its opposite, hypofunction) represents mildly deleterious processes and ultimately contributes to the deleteriome. In the end, many existing aging theories are right in the sense that they correctly point to a particular aspect of aging, but they are also incomplete in other aspects.

Integration of aging theories can be terrifically useful. Considering imperfectness, the deleteriome, and the role of genetics, environment, and stochasticity, as opposed to gene‐/process‐centric views, would shun conclusions on the key roles of specific processes in causing aging, which currently represent the bulk of publications in the field. Indeed, when a particular gene or process is altered thereby affecting lifespan, the molecular basis may be an altered metabolism, which translates to a different set of deleterious processes and results in a different deleteriome. Such experiments can teach us about the processes that may affect lifespan, but they tell us little if anything about the nature of aging. We must then ask: If this aspect of aging is incomplete or even irrelevant, what is left? What do we actually understand about the molecular basis of aging and the control of lifespan? To some, the answer may be disheartening, given the difficulties in experimental analysis of these questions. Yet, it is important to abandon comfortable single gene‐centric and single process‐centric thinking in favor of integrative concepts that may help to ask the right questions in future aging research.

## Funding

Supported by grants from the National Institute on Aging.

## Conflict of interest

None declared.

## References

[acel12480-bib-0001] Ackerman D , Gems D (2012) The mystery of *C. elegans* aging: an emerging role for fat: distant parallels between *C. elegans* aging and metabolic syndrome? BioEssays 34, 466. 22371137 10.1002/bies.201100189

[acel12480-bib-0002] Ackermann M , Chao L , Bergstrom CT , Doebeli M (2007) On the evolutionary origin of aging. Aging Cell 6, 235–244. 17376147 10.1111/j.1474-9726.2007.00281.x PMC2049046

[acel12480-bib-0003] Avanesov AS , Ma S , Pierce KA , Yim SH , Lee BC , Clish CB , Gladyshev VN (2014) Age‐ and diet‐associated metabolome remodeling characterizes the aging process driven by damage accumulation. eLife 3, e02077. 24843015 10.7554/eLife.02077 PMC4003482

[acel12480-bib-0004] Blagosklonny MV (2008) Aging: ROS or TOR. Cell Cycle 7, 3344. 18971624 10.4161/cc.7.21.6965

[acel12480-bib-0005] Budovskaya YV , Wu K , Southworth LK , Jiang M , Tedesco P , Johnson TE , Kim SK (2008) An elt‐3/elt‐5/elt‐6 GATA transcription circuit guides aging in *C. elegans* . Cell 134, 291–303. 18662544 10.1016/j.cell.2008.05.044 PMC4719053

[acel12480-bib-0006] Charlesworth B (1994) Evolution in Age‐Structured Populations. Cambridge: Cambridge University Press.

[acel12480-bib-0007] Coelho M , Dereli A , Haese A , Kühn S , Malinovska L , DeSantis ME , Shorter J , Alberti S , Gross T , Tolić‐Nørrelykke IM (2013) Fission yeast does not age under favorable conditions, but does so after stress. Curr. Biol. 23, 1844–1852. 24035542 10.1016/j.cub.2013.07.084 PMC4620659

[acel12480-bib-0008] Evans SN , Steinsaltz D (2007) Damage segregation at fissioning may increase growth rates: a superprocess model. Theor. Popul. Biol. 71, 473–490. 17442356 10.1016/j.tpb.2007.02.004 PMC2430589

[acel12480-bib-0009] Fang X , Seim I , Huang Z , Gerashchenko MV , Xiong Z , Turanov AA , Zhu Y , Lobanov AV , Fan D , Yim SH , Yao X , Ma S , Yang L , Lee SG , Kim EB , Bronson RT , Sumbera R , Buffenstein R , Zhou X , Krogh A , Park TJ , Zhang G , Wang J , Gladyshev VN (2014) Adaptations to a subterranean environment and longevity revealed by the analysis of mole rat genomes. Cell Rep. 8, 1354–1364. 25176646 10.1016/j.celrep.2014.07.030 PMC4350764

[acel12480-bib-0010] Finch CE (1990) Longevity, Senescence and the Genome. Chicago: University of Chicago Press.

[acel12480-bib-0011] Fushan AA , Turanov AA , Lee SG , Kim EB , Lobanov AV , Yim SH , Buffenstein R , Lee SR , Chang KT , Rhee H , Kim JS , Yang KS , Gladyshev VN (2015) Gene expression defines natural changes in mammalian lifespan. Aging Cell 14, 352–365. 25677554 10.1111/acel.12283 PMC4406664

[acel12480-bib-0012] Gavrilov LA , Gavrilova NS (2001) The reliability theory of aging and longevity. J. Theor. Biol. 213, 527–545. 11742523 10.1006/jtbi.2001.2430

[acel12480-bib-0013] Gladyshev VN (2012) On the cause of aging and control of lifespan: heterogeneity leads to inevitable damage accumulation, causing aging; control of damage composition and rate of accumulation define lifespan. BioEssays 34, 925. 22915358 10.1002/bies.201200092 PMC3804916

[acel12480-bib-0014] Gladyshev VN (2013) The origin of aging: imperfectness‐driven non‐random damage defines the aging process and control of lifespan. Trends Genet. 29, 506–512. 23769208 10.1016/j.tig.2013.05.004 PMC3804915

[acel12480-bib-0015] Gladyshev VN (2014) The free radical theory of aging is dead. Long live the damage theory!. Antioxid. Redox Signal. 20, 727–731. 24159899 10.1089/ars.2013.5228 PMC3901353

[acel12480-bib-0016] Golubev AG (1996) The other side of metabolism. Biokhimiia 61, 2018–2039. 9004862

[acel12480-bib-0017] Haldane JBS (1941) New Paths in Genetics. London: Allen & Unwin.

[acel12480-bib-0018] Hamilton WD (1966) The moulding of senescence by natural selection. J. Theor. Biol. 12, 12–45. 6015424 10.1016/0022-5193(66)90184-6

[acel12480-bib-0019] Hannum G , Guinney J , Zhao L , Zhang L , Hughes G , Sadda S , Klotzle B , Bibikova M , Fan JB , Gao Y , Deconde R , Chen M , Rajapakse I , Friend S , Ideker T , Zhang K (2013) Genome‐wide methylation profiles reveal quantitative views of human aging rates. Mol. Cell 49, 359–367. 23177740 10.1016/j.molcel.2012.10.016 PMC3780611

[acel12480-bib-0020] Harman D (1956) Aging: a theory based on free radical and radiation chemistry. J. Gerontol. 11, 298. 13332224 10.1093/geronj/11.3.298

[acel12480-bib-0021] Hoffman JM , Soltow QA , Li S , Sidik A , Jones DP , Promislow DE (2014) Effects of age, sex, and genotype on high‐sensitivity metabolomic profiles in the fruit fly, *Drosophila melanogaster* . Aging Cell 13, 596–604. 24636523 10.1111/acel.12215 PMC4116462

[acel12480-bib-0022] Horvath S (2013) DNA methylation age of human tissues and cell types. Genome Biol. 14, R115. 24138928 10.1186/gb-2013-14-10-r115 PMC4015143

[acel12480-bib-0023] Jones OR , Scheuerlein A , Salguero‐Gómez R , Camarda CG , Schaible R , Casper BB , Dahlgren JP , Ehrlén J , García MB , Menges ES , Quintana‐Ascencio PF , Caswell H , Baudisch A , Vaupel JW (2014) Diversity of ageing across the tree of life. Nature 505, 169–173. 24317695 10.1038/nature12789 PMC4157354

[acel12480-bib-0024] Kaya A , Lobanov AV , Gladyshev VN (2015) Evidence that mutation accumulation does not cause aging in *Sacharomyces cerevisiae* . Aging Cell 14, 366–371. 25702753 10.1111/acel.12290 PMC4406665

[acel12480-bib-0025] Kirkwood TB (1977) Evolution of ageing. Nature 270, 301. 593350 10.1038/270301a0

[acel12480-bib-0026] Kirkwood TB , Austad SN (2000) Why do we age? Nature 408, 233–238. 11089980 10.1038/35041682

[acel12480-bib-0027] Koonin EV (2011) The Logic of Chance. Upper Saddle River, NJ: FT Press Science.

[acel12480-bib-0028] Lemaître JF , Gaillard JM , Pemberton JM , Clutton‐Brock TH , Nussey DH (2014) Early life expenditure in sexual competition is associated with increased reproductive senescence in male red deer. Proc. Biol. Sci. 281, 1792. 10.1098/rspb.2014.0792 PMC4150313 25122226

[acel12480-bib-0029] Longo VD , Mitteldorf J , Skulachev VP (2005) Programmed and altruistic ageing. Nat. Rev. Genet. 6, 866–872. 16304601 10.1038/nrg1706

[acel12480-bib-0030] López‐Otín C , Blasco MA , Partridge L , Serrano M , Kroemer G (2013) The hallmarks of aging. Cell 153, 1194–1217. 23746838 10.1016/j.cell.2013.05.039 PMC3836174

[acel12480-bib-0031] Ma S , Yim SH , Lee SG , Kim EB , Lee SR , Chang KT , Buffenstein R , Lewis KN , Park TJ , Miller RA , Clish CB , Gladyshev VN (2015) Organization of the mammalian metabolome according to organ function, lineage specialization, and longevity. Cell Metab. 22, 332–343. 26244935 10.1016/j.cmet.2015.07.005 PMC4758382

[acel12480-bib-0032] de Magalhaes JP (2012) Programmatic features of aging originating in development: aging mechanisms beyond molecular damage? FASEB J. 26, 4821. 22964300 10.1096/fj.12-210872 PMC3509060

[acel12480-bib-0033] Medawar PB (1952) An Unsolved Problem of Biology. London: HK Lewis.

[acel12480-bib-0034] Moorad JA , Promislow DE (2008) A theory of age‐dependent mutation and senescence. Genetics 179, 2061–2073. 18660535 10.1534/genetics.108.088526 PMC2516080

[acel12480-bib-0035] O'Brien DM , Min KJ , Larsen T , Tatar M (2008) Use of stable isotopes to examine how dietary restriction extends *Drosophila* lifespan. Curr. Biol. 18, R155–R156. 18302914 10.1016/j.cub.2008.01.021

[acel12480-bib-0036] Orgel LE (1973) Ageing of clones of mammalian cells. Nature 243, 441. 4591306 10.1038/243441a0

[acel12480-bib-0037] Rose MR (1991) Evolutionary Biology of Aging. New York: Oxford University Press.

[acel12480-bib-0038] Seim I , Fang X , Xiong Z , Lobanov AV , Huang Z , Ma S , Feng Y , Turanov AA , Zhu Y , Lenz TL , Gerashchenko MV , Fan D , Yim SH , Yao X , Jordan D , Xiong Y , Ma Y , Lyapunov AN , Chen G , Kulakova OI , Sun Y , Lee SG , Bronson RT , Moskalev AA , Sunyaev SR , Zhang G , Krogh A , Wang J , Gladyshev VN (2013) Genome analysis reveals insights into physiology and longevity of the Brandt's bat *Myotis brandtii* . Nat. Commun. 4, 2212. 23962925 10.1038/ncomms3212 PMC3753542

[acel12480-bib-0039] Sinclair DA , Guarente L (1997) Extrachromosomal rDNA circles–a cause of aging in yeast. Cell 91, 1033–1042. 9428525 10.1016/s0092-8674(00)80493-6

[acel12480-bib-0040] Skulachev VP (1997) Aging is a specific biological function rather than the result of a disorder in complex living systems: biochemical evidence in support of Weismann's hypothesis. Biochemistry (Mosc) 62, 1191–1195. 9467841

[acel12480-bib-0041] Somel M , Guo S , Fu N , Yan Z , Hu HY , Xu Y , Yuan Y , Ning Z , Hu Y , Menzel C , Hu H , Lachmann M , Zeng R , Chen W , Khaitovich P (2010) MicroRNA, mRNA, and protein expression link development and aging in human and macaque brain. Genome Res. 20, 1207–1218. 20647238 10.1101/gr.106849.110 PMC2928499

[acel12480-bib-0042] Stearns SC (1992) The Evolution of life Histories. Oxford: Oxford University Press.

[acel12480-bib-0043] Vijg J , Campisi J (2008) Puzzles, promises and a cure for ageing. Nature 454, 1065. 18756247 10.1038/nature07216 PMC2774752

[acel12480-bib-0044] Watve M , Parab S , Jogdand P , Keni S (2006) Aging may be a conditional strategic choice and not an inevitable outcome for bacteria. Proc. Natl Acad. Sci. USA 103, 14831–14835. 17001004 10.1073/pnas.0606499103 PMC1595437

[acel12480-bib-0045] Wensink MJ , Wrycza TF , Baudisch A (2014) No senescence despite declining selection pressure: Hamilton's result in broader perspective. J. Theor. Biol. 347, 176–181. 24316386 10.1016/j.jtbi.2013.11.016

[acel12480-bib-0046] Williams GC (1957) Pleiotropy, natural selection, and the evolution of senescence. Evolution 11, 398.

[acel12480-bib-0047] Yin D (2016) The essential mechanisms of aging: what have we learnt in ten years? Curr. Top. Med. Chem. 16, 503–510. 26268334 10.2174/1568026615666150813142854

